# The ubiquitous experience of alcohol industry involvement in science: Findings from a qualitative interview study

**Published:** 2022-03-01

**Authors:** Gemma Mitchell, Jim McCambridge

**Affiliations:** 1 Department of Health Sciences, University of York, York, UK

## Abstract

**Objective:**

There is little formal study of alcohol industry involvement in science, despite longstanding concerns about various activities, and broader evidence of corporate manipulation of research. We aimed to explore the experiences of researchers who had no relationship with the alcohol industry, including how industry involvement in alcohol science more broadly had impacted their research work.

**Method:**

Qualitative, semi-structured interview study with senior researchers working on alcohol policy-relevant topics who had not received any form of payment from the alcohol industry, or performed any unpaid work for alcohol industry companies, or organisations they have created (n=14). A thematic analysis of transcripts using NVivo software was undertaken.

**Results:**

Despite not having worked with industry, contact with industry was nonetheless unavoidable for these alcohol researchers. This was particularly the case at conferences and policy-related events, which formed a key strand of broader industry surveillance of the research field, including individuals in the research community, and research outputs. Monitoring of the research community at conferences also afforded opportunities for informal relationship-building and attempts to exercise influence. Where research findings were contrary to business interests, surveillance served as a platform for interventions of various kinds, including issuing legal threats.

**Conclusions:**

The alcohol industry extensively monitors research and researchers. Researchers who study the alcohol industry are targeted in particular, both covertly and overtly. Researchers experience the alcohol industry as ubiquitous in alcohol policy-related research, with conferences and policy-related events key venues for both relationship building and surveillance.

## Introduction

Concerns about alcohol industry conflicts of interests in relation to science are extensive (Babor, 2009; McCambridge & Mialon, 2018) and informed by numerous commentaries, editorials, and reviews on the subject (for example, Babor & Robaina, 2013; Jernigan, 2012; Stenius & Babor, 2010). One concern relates to the motivations underlying any alcohol industry scientific activity, which stems from apparent similarities with wider corporate attempts to manipulate or ‘bend’ science to suit commercial interests across a range of health and environmental subjects (McGarity & Wagner, 2008; Michaels, 2020; Nestle, 2018; Oreskes & Conway, 2010; White & Bero, 2010). Other concerns analysed in a dedicated systematic review relate to transgressions of basic academic norms, including not adhering to peer review processes, and the ways in which alcohol industry involvement in science may bias the literature, for example industry funding skewing research agendas (McCambridge & Mialon, 2018). Despite the longstanding nature of these concerns, there is little formal study of alcohol industry scientific activity, even though it is extensive (Golder et al., 2020). Wider evidence on tobacco industry activity can provide relevant insights, because the tobacco and alcohol industries are deeply connected, for example through co-ownership of companies (Bond et al., 2010; Hawkins & McCambridge, 2018), and there is extensive evidence on the instrumental and public relations benefits for tobacco companies arising from involvement within the sphere of peer-reviewed science (Bero, 2005).

There is a global consensus within public health sciences that a whole population approach, including regulatory measures, is the most effective way of reducing alcohol harms (World Health Organization, 2018). Yet most national alcohol policies, where they exist, do not reflect this consensus, eschewing whole population approaches that would impinge on the commercial activities of the alcohol industry (McCambridge, Kypri, et al., 2019). There is an emerging literature indicating that the alcohol industry is extensively involved in alcohol policy making, using a range of sophisticated strategies that have much in common with tobacco industry policy activity (McCambridge, Coleman, et al., 2019; McCambridge et al., 2018; Savell et al., 2016). In order to be influential in the realm of alcohol policy, the alcohol industry requires a body of publications to legitimise its claims. Visible attempts by industry actors to influence policy reveal that they fundamentally misrepresent and misuse the evidence-base (McCambridge et al., 2013; Rossow & McCambridge, 2019; Stafford et al., 2020). Yet, little is known about the extent to which the alcohol industry influences the conduct of science itself (McCambridge & Hartwell, 2015; McCambridge & Mialon, 2018).

Two recent episodes highlight the importance of studying this topic. The first is the largely-industry funded $100 million Moderate Alcohol and Cardiovascular Health (MACH) trial, which was terminated due to a biased trial design co-produced by public sector research funding officials, researchers, and alcohol industry executives (Mitchell et al., 2020). The second occurred when a study in Canada examining the introduction of cancer warning labels on alcohol containers was shut down due to industry interference (Stockwell et al., 2020).

Informed by the concerns raised in the literature outlined above, we undertook an interview study of alcohol researchers (n = 37/44 invited), with 23 participants having some form of relationship with industry (see below for definitions). It transpired that 14 researchers had not worked with industry, and in this study we examine their experiences, including the impacts of alcohol industry involvement in science on their research work. This study is thus nested within the larger interview study. Elsewhere we report on the impacts of early career industry research funding (Mitchell & McCambridge, in press) and senior researchers who worked with social aspects organisations once their careers were established (Mitchell & McCambridge, 2021).

## Method

To construct our research questions, we drew on the systematic review of concerns raised in the peer-reviewed literature about alcohol industry involvement in science (McCambridge & Mialon, 2018), as well as the extensive evidence available on tobacco industry involvement in science (for example, Bero, 2005; Landman & Glantz, 2009). We also utilised the sociology of knowledge and science and technology studies (STS) literatures, applying a qualitative approach to this exploratory study where the interest lies in the values and meanings researchers attach to their scientific work or ‘practice’ (Latour, 1987; Latour & WooIgar, 1986; Pickering, 1992). Thus, we are mindful of the diversity of ‘epistemic cultures’ within what might be referred to as the scientific community (Knorr-Cetina, 1999); the different forms of knowledge used by researchers (Collins, 2010); and how social processes play a part in how disagreements between scientists or ‘scientific controversies’ are resolved (Collins, 1974, 2004). By focusing on the work involved in constructing scientific knowledge, rather than viewing scientific knowledge as something ‘out there’ and separate to human activity, we were able to study something tangible that is rooted in social interactions in order to explore the often hidden activity of the alcohol industry.

We purposively sampled senior researchers based primarily on the second author’s knowledge of their standing in the field, almost all of whom were based at a university. We recruited across Europe, North America, and Australasia, which is where those researchers identified were based, and only sampled researchers who publish in the English language. To explore how the alcohol industry has engaged with non-industry funded research and researchers, we recruited researchers who, to our knowledge, had not received any form of payment (including research funding, honorariums, and expenses) from any alcohol industry-related body or performed any work (paid or unpaid) for the alcohol industry (n=14). This includes seven researchers who had studied industry at some point in their career. The interviewees were based in the US (n=5), the UK (n=4), mainland Europe (n=4) and Australia (n=1).We tried to recruit across genders, although there was no formal sampling requirement; nine researchers were male, five female. All participants in the parent interview study were identified because their work was judged in some way policy relevant; not that they were alcohol policy researchers but that their studies may be cited in policy documents. We in no way claim this group is representative of all researchers working on alcohol policy-relevant topics who do not have relationships with the alcohol industry.

Semi-structured interviews were completed via video call or telephone, and the length of interviews ranged from 30 minutes to 2 hours, averaging 60 - 75 minutes. Researchers were first asked about their career development, then the nature of any contacts with the alcohol industry, factors informing their decision-making regarding whether or not to work with the alcohol industry, and their perceptions of a number of concerns about alcohol industry involvement in science summarised in a systematic review (McCambridge & Mialon, 2018). Whereas the first author is new to the field, the second author is a researcher who has studied this topic, which led to moments of ‘insider/outsider’ dynamics during the interviews (Kusow, 2003).

Data were analysed using a form of reflexive thematic analysis (Braun & Clarke, 2006; Braun et al., 2019), with each transcript read prior to initial coding using NVivo software by the first author. Coding developed iteratively rather than via a fixed codebook at the start of the process. Themes were generated alongside and subsequent to the coding, and the first author referred to the literature throughout to make sense of the data (Timmermans & Tavory, 2012). The second author read the transcripts and supported the first author throughout the analysis process. The study received ethical approval from the University of York Health Sciences Research Governance Committee. We have removed all identifying information about the participants from the quotes provided below, and do not use pseudonyms to further protect researcher anonymity. We have directly quoted ten of the 14 participants in this paper; the quotes are representative of the views and experiences identified in the dataset as a whole.

## Results

Despite not having worked with industry, contact with industry was nonetheless unavoidable for these researchers. This contact took two main forms. The first took place via industry attendance at, and participation in, research conferences and policy-related events. Industry personnel encountered by researchers included trade association representatives, paid consultants, and representatives from social aspects organisations. Interviewees reported having varying perceptions of the visibility of industry actors at conferences and other events, and varying levels of contact with them. Alongside this monitoring activity, ten of the 14 researchers reported a second type of contact, where industry were engaged in more targeted surveillance of their research work (and for some, interventions with them).

### Routine contacts with industry at conferences

Researchers reported that both research conferences and alcohol policy-related events were key spaces in which they regularly came into contact with the alcohol industry. Attendance at these events allowed a range of alcohol industry actors to access extensive information about research and researchers.

#### Alcohol research conferences

Contact with industry personnel at scientific conferences is commonplace, and was largely regarded as part of the territory of being an alcohol researcher. Not only are alcohol industry actors present at research conferences, but a number of these events have been sponsored by alcohol industry organisations. Specific events (both industry-sponsored and non-industry sponsored) were identified as having free alcohol and lending themselves to heavy drinking, although one researcher noted differences between the two types:

This sounds trivial: I don’t think it is though. One of the big differences is that with industry involvement, you’ve got a lot more sponsored lunches, open bars, and a kind of higher level of expenses on what we might [call] hospitality functions than you might get otherwise.

Interpersonal contacts take place at both scientific conferences and alcohol policy-related events (see below), and can occur at any career stage, including during early career socialisation:

I presented some work [at early career stage]…and a very nice woman, probably a bit older than me was encouraging of the work. And I hadn't really thought much about who she represented. But she started encouraging me…if you look on the bright side a little bit, not to be always telling a bad news story. And I thought where did that come from? What's that about?…It was the beginning of a long series of interactions with the same organisation…

During the formal parts of such events, both senior and junior industry representatives would at times take part in discussion panels, as well as asking questions following presentations.

Interactions with industry also take place at the boundaries of organised activities, for example during conference breaks:

[A trade association representative] just came up to me, he was very keen to talk…And similarly at [a different conference] I bumped into [a major alcohol producer representative] in the toilets. And he had a couple of comments on my presentation.

Contacts were also reported prior to the event itself:

One time I was on a plane on my way to a conference and the woman who was sitting across the aisle saw that I was working on some slides and started a conversation and she was a representative from [a trade association]… We had a conversation and then she gave me a ride to the conference after we landed.

#### Taken-for-granted: a lack of clarity associated with policy-related events

Because much clinical, population health, and social science research on alcohol is highly policy-relevant, policy-related events are organised by a variety of stakeholders involved in informing policy with scientific evidence. These include events organised by governments, nongovernmental and intergovernmental organisations, not-for-profit organisations, charities, think tanks, and private companies. Industry actors tend to be more involved in these kinds of meetings; again, this was seen as unremarkable. The extent of industry presence at some events can nonetheless be surprising. One researcher, for example, reported a particular experience where it was not at all clear when they initially accepted an invitation to present at an event that industry were involved. This situation is not uncommon and creates additional work for researchers, who reported finding it challenging to find out in advance how involved industry may be in policy-related events. When they did attend such events, researchers also reported that they had participated in panels with alcohol industry actors:

We were invited at some point to join a panel sitting up out the front, and I was seated next to [an] industry executive and I made some comments to the effect that nothing against this person personally, but they shouldn't be here… well, there was condemnation at that remark by some people in the audience. And so the point is that they were actually involved. They were seen to be integral to the discussion. Rather than observers.

Researchers reported many intricacies involved in decision-making about their contact with industry at events. One researcher gave an example of accepting an invitation to attend an alcohol industry-funded group’s ‘workshop’ on a specific topic, along with other researchers doing similar work. After being invited to a second workshop, discussion with colleagues led the researcher to decline, due to wariness of building a ‘partnership’ with the industry group.

#### Amiable conversations

Researchers reported that most interactions at both research conferences and policy-related events were amiable. Researchers noted being encouraged to look at particular issues or in particular ways, and were congratulated on work on topics that did not conflict with commercial interests. Occasionally, such informal conversations with alcohol industry actors provided glimpses into the biased nature of alcohol industry scientific activity:

We [researcher and trade organisation employee] talked about a systematic review that she was working on funded by the industry of things that reduced alcohol harms. And she asked me if I thought that this was a good thing for them to be working on? And then I asked her whether taxation was one of the items that was going to be in her systematic review? And her view was that that wasn’t so relevant because the evidence was so mixed.

The evidence on taxation is strong, and such thinking betrays a lack of appreciation of the nature of a systematic review. Both junior and senior industry personnel participate in conferences, with junior industry representatives seen to be lacking public health knowledge. More knowledgeable, senior representatives, however, can use informal interactions to elicit useful information:

I think it was a guy from [global alcohol producer] came up to me after my presentation and sort of asked [a] question which seemed innocuous at the time…And I gave a straight answer. That answer then kept cropping up [in critiques of the researcher’s work].

Senior figures include ‘revolving door’ individuals who have moved from public sector organisations such as universities to employment with industry and are well placed to build and sustain relationships. Informal conversations can be used as information gathering opportunities by researchers, as well as the other way round, with researchers attempting to get to know individual industry actors and understand their motivations and activity. Interviewees had varying perceptions of the visibility of industry actors at conferences and other events, and varying levels of contact with them, with some researchers seeking to avoid. Seemingly inconsequential conversations with alcohol industry actors nonetheless appear part of wider industry monitoring of researchers and their outputs.

### Alcohol industry surveillance of researchers, and interventions for some

Ten of the 14 researchers reported some form of direct surveillance of their research activity, and this was the case for all seven who studied the industry.

#### Targeted surveillance

Surveillance of research and researchers takes various forms, the least intrusive of which is extensive monitoring of the scientific literature, which can lead on to industry responses to research outputs. Letters from alcohol industry actors in response to journal articles and opinion pieces in the media were commonplace for these interviewees, and these may also be sent directly to the researcher. Researchers also reported being named in blog posts, articles in the alcohol trade press, and media interventions more broadly, as well as in comments on social media. Responses to research activity also included more lengthy written critiques commissioned by the alcohol industry.

Overall, these responses had an appearance of engaging with the science, and sometimes reflected a high level of scientific expertise, but often misrepresented the researcher’s findings. Sometimes such content made major scientific errors. The surveillance thus morphed into intervening in the processes of research production and/or dissemination. This could lead to time-consuming decision-making about whether and how to respond, with increased uncertainty evident at the early career stage:

They clearly had a skilled, knowledgeable person [with scientific] training who wrote a long letter to me seeking to criticise or questioning the study…And I began very conscientiously to write a detailed response about how they were mistaken. That we'd done X, Y, and Z, and in fact those decisions in our analysis were justified and conservative even, blah-blah-blah. And my advisors or seniors at the time said oh, don't worry about it too much. It's completely standard, everything we publish, they write to us. They write routinely. So that was an eye-opener.

Additionally, surveillance of research presented at conferences can take place without actual attendance:

[An alcohol company] had noticed that I had sent in an abstract to a conference…And I got a letter from [the alcohol company] afterwards. They said, we read your abstract and we want to know what your results are about, or we would like to have your results…if I read between the lines, it was sort of like, we’re keeping our eye on you.

Three researchers reported individual meetings with alcohol producers or trade associations who approached them, and were of the view that this was one way for industry to monitor researchers and their scientific outputs. With regards to researchers getting information from the alcohol industry, a different picture emerged. Where researchers or their colleagues had attended meetings or had discussions with alcohol industry actors with the aim of gaining access to valuable industry data, every attempt failed.

#### Targeting individuals, both covertly and overtly

Seven researchers, six of whom studied the industry, reported additional alcohol industry attention that went beyond surveillance. This included alcohol industry actors contacting research funding organisations and/or academic institutions, which may be done overtly or covertly:

They will contact actual funders who might have funded you, and so on. And that’s part of this…sort of chilling effect. It’s to make you appear a problematic person.

This can, again, lead to time-consuming additional tasks for researchers:

We were essentially audited. An outside expert was brought in. Everything we had produced was gone over with a fine toothcomb …we got through it, we were not undone, but it certainly slowed us down.

‘Revolving door’ individuals – former academic colleagues who moved on to work with the alcohol industry, including alcohol industry-funded groups – are well placed to undertake surveillance, because they can use their scientific networks to gather information about researchers:

The first thing that person did was to begin calling his former contacts in the scientific community and asking them about people like me and whether they had any information as to why I was preoccupied with studying the industry. But it seemed like his job, or one of his assignments, was to try to use his network contacts to get information about people like me.

One researcher reported that alcohol industry actors had contacted research participants during the data collection phase of a research project. The researcher found this out via the research participants.

Four researchers who studied the industry reported that their integrity as researchers was called into question, or they were attacked by the industry in the media as part of responses to research findings that conflicted with commercial interests. Surveillance thus serves as a platform for intimidation designed to impede policy-relevant research that produces findings contrary to business interests, particularly on the alcohol industry itself. These interventions were particularly associated with research that was widely disseminated in the public domain. Academic norms of transparency are such that some researchers who have been targeted by industry nevertheless respond positively to industry requests for information:

I've always had an open door policy. I'll talk to anybody in the industry who wants to talk to me.

Industry responses to research that does not support commercial interests range from relatively subtle efforts to undermine reputations via critiques in journals and other academic institutions, in the media and elsewhere in the public domain, through to threats of legal actions. Three researchers, each of whom have studied the alcohol industry, had received legal threats from alcohol industry actors over the course of their career. Researchers noted that legal threats are issued quickly, for example after comments made in the media:

When I said something rude about [alcohol company] in a newspaper I got a lawyer’s letter the next day. So that was a real wake-up call. They were watching everything.

Legal threats can also involve institutions as well as individuals, and be quite complex to address. The onerous nature of the costs involved may also provide reasons why the institutions concerned may proceed cautiously:

All the lawyers who've looked at this, the various parties’ lawyers have said nothing we've written is defamatory. But it could still end up in court. So it's not that anyone thinks what we've done is wrong, it's that they think they may have to defend it. And it's the risk of having to defend it that they're protecting against, not the risk of losing. And there's a slim risk of losing but that's sufficient to make them quite cautious… it just makes me realise that we're more vulnerable than I thought. The kinds of protections you might have hoped for are just not there.

The distinct challenges posed in doing alcohol policy-relevant research may be unexpected when researchers enter the field. There was widespread agreement that alcohol industry involvement in research posed important issues that needed to be more fully discussed in the scientific community in collegiate ways. For many, this was predicated on having a clearer understanding of the risks this posed to research.

## Discussion

A chat over lunch at a conference, or bumping into someone in a corridor on the way to present a paper may appear inconsequential to the individual researcher. Our data imply, however, that these apparently minor interactions are part of a broader pattern of alcohol industry scientific activity in which researchers are observed and relationships are built. Scientific conferences and related events are thus an important gateway into science for industry actors. This activity also forms part of industry’s broader monitoring of science, with researchers who study topics that are highly relevant to industry, or indeed industry itself, being particularly exposed to more targeted surveillance and interventions that go beyond surveillance, including harassment and legal threats. Utilising qualitative methods underpinned by a sociological framework has enabled us to identify these (largely invisible) mechanisms of industry influence on scientific processes.

There are longstanding concerns about alcohol industry involvement in science, and this study, the first of its kind, provides an empirical foundation for future studies. The encouragingly high response rate, combined with in-depth reflections covering research careers spanning decades, provides a rich source of data on the everyday experience of doing alcohol-policy relevant research. Although researchers were based in several different countries, there is a reliance on high-income Anglophone countries in our sample. The researcher experience of industry in low and middle-income countries also requires attention. That the sample is based on the second author’s knowledge of the field is another clear limitation, as is only including researchers who publish in English. The 14 researchers studied here were senior researchers whose reflections were largely based on the past, sometimes distantly so; hence the somewhat historical nature of these retrospective accounts should be borne in mind in interpreting these data. This is a controversial and sensitive topic to discuss; as a consequence, researchers will have varied in their willingness to discuss their experiences.

The alcohol industry appears primarily involved in science in order to influence policy (McCambridge & Mialon, 2018; Rossow & McCambridge, 2019). Our findings indicate that senior industry actors are prominent at policy-related events. Such events give industry the ability to be actively involved in shaping consideration of how evidence may inform policy making, interfering with this process to advance commercial interests. This finding, combined with evidence on industry attendance at scientific meetings, means there is a need for transparency in decision-making about the organisation of these types of events so as not to compromise scientific integrity. Further study will be helpful to understanding the roles of conferences, as well as other venues and mechanisms of surveillance, relationship building, and the targeting of researchers.

An overarching concern raised in earlier discussions of alcohol industry involvement in science is superficial adherence to scientific procedures, at best, alongside a lack of genuine engagement with the scientific process (McCambridge & Mialon, 2018). Our findings bear this out in the form of covert surveillance, misrepresentation of findings, and personal attacks on individual integrity. The extensive surveillance and harassment of researchers whose work does not support commercial interests identified here, particularly of those who study the industry, warrants protection of researchers. This kind of activity shares similarities with other industries (Landman & Glantz, 2009; McGarity & Wagner, 2008; Michaels, 2020; Oreskes & Conway, 2010; White & Bero, 2010), with one recent alcohol study providing an example of the serious disruption to research such activity can cause (Stockwell, et al., 2020). Yet, there are still today few protections, rules, or guidelines for researchers on how to deal with such occupational hazards. Researchers have been left to navigate these challenges as individuals, informed only by anecdotes, shared concerns, and personal experiences. Discussion of how the alcohol research community addresses these challenges, including on developing guidance, advice and support for early career researchers, is long overdue. The alcohol research community is not alone. University research integrity training also needs to be developed to address other corporate vested interests in research.

Our findings corroborate existing concerns about the various mechanisms industry use to 1) enter into the scientific process; 2) build relationships with researchers; 3) monitor research and researchers; and 4) attack research (and individuals) where the findings do not support commercial interests (McCambridge & Mialon, 2018). Researchers often reported uncertainty about how to respond to industry activity, particularly at the early career stage, and they ‘picked up’ various strategies to respond to this via informal conversations with supervisors and other colleagues over the course of their career. Industry actors appear adept at managing informal social interactions that are part of the ‘practice’ of science (Pickering, 1992), with senior industry figures able to draw upon tacit knowledge of how research works. These findings may generalise to other domains of corporate science activity relevant to public health, and demonstrate how social scientists can contribute to broadening understanding of such processes.

This study begs important questions about the extent to which scientific norms and rules that govern researcher behaviour have been subtly shaped by the ubiquitous nature of industry involvement in alcohol research over time. It invites questions about what else industry actors have done in alcohol science, and to what extent is what we know about alcohol, health, and society shaped by the ubiquitous nature of alcohol industry involvement in science? Answering such questions will benefit from situating what has been found for alcohol in relation to the approaches used to study the conduct of science and the manipulation of science by corporate interests more broadly.

## References

[R1] Babor TF (2009). Alcohol research and the alcoholic beverage industry: Issues, concerns and conflicts of interest. Addiction.

[R2] Babor TF, Robaina K (2013). Public health, academic medicine, and the alcohol industry’s corporate social responsibility activities. American Journal of Public Health.

[R3] Bero L (2005). Tobacco industry manipulation of research. Public Health Reports.

[R4] Bond L, Daube M, Chikritzhs T (2010). Selling addictions: Similarities in approaches between big tobacco and big booze. Australasian Medical Journal.

[R5] Braun V, Clarke V (2006). Using thematic analysis in psychology. Qualitative Research in Psychology.

[R6] Braun V, Clarke V, Hayfield N, Terry G, Liamputtong P (2019). Handbook of research methods in health social sciences.

[R7] Collins HM (1974). The tea set: Tacit knowledge and scientific networks. Science Studies.

[R8] Collins HM (2004). Gravity's shadow: The search for gravitational waves.

[R9] Collins HM (2010). Tacit and explicit knowledge.

[R10] Golder S, Garry J, McCambridge J (2020). Declared funding and authorship by alcohol industry actors in the scientific literature: A bibliometric study. European Journal of Public Health.

[R11] Hawkins B, McCambridge J (2018). Can internal tobacco industry documents be useful for studying the uk alcohol industry?. BMC Public Health.

[R12] Jernigan DH (2012). Global alcohol producers, science, and policy: The case of the international center for alcohol policies. American Journal of Public Health.

[R13] Knorr-Cetina K (1999). Epistemic cultures: How the sciences make knowledge.

[R14] Landman A, Glantz SA (2009). Tobacco industry efforts to undermine policy-relevant research. American Journal of Public Health.

[R15] Latour B (1987). Science in action: How to follow scientists and engineers through society.

[R16] Latour B, WooIgar S (1986). Laboratory life: The construction of scientific facts.

[R17] McCambridge J, Coleman R, McEachern J (2019). Public health surveillance studies of alcohol industry market and political strategies: A systematic review. Journal of Studies on Alcohol and Drugs.

[R18] McCambridge J, Hartwell G (2015). Has industry funding biased studies of the protective effects of alcohol on cardiovascular disease? A preliminary investigation of prospective cohort studies. Drug and Alcohol Review.

[R19] McCambridge J, Hawkins B, Holden C (2013). Industry use of evidence to influence alcohol policy: A case study of submissions to the 2008 scottish government consultation. PLOS Medicine.

[R20] McCambridge J, Kypri K, Sheldon TA, Madden M, Babor TF (2019). Advancing public health policy making through research on the political strategies of alcohol industry actors. Journal of Public Health.

[R21] McCambridge J, Mialon M (2018). Alcohol industry involvement in science: A systematic review of the perspectives of the alcohol research community. Drug and Alcohol Review.

[R22] McCambridge J, Mialon M, Hawkins B (2018). Alcohol industry involvement in policymaking: A systematic review. Addiction.

[R23] McGarity TO, Wagner WE (2008). Bending science: How special interests corrupt public health research.

[R24] Michaels D (2020). The triumph of doubt: Dark money and the science of deception.

[R25] Mitchell G, Lesch M, McCambridge J (2020). Alcohol industry involvement in the moderate alcohol and cardiovascular health trial. American Journal of Public Health.

[R26] Mitchell G, McCambridge J (2021). Recruitment, risks, rewards and regrets: Senior researcher reflections on working with alcohol industry social aspects organisations. Drug and Alcohol Review.

[R27] Mitchell G, McCambridge J The ‘snowball effect’: Short and long-term consequences of early career alcohol industry research funding. Addiction, Research & Theory.

[R28] Nestle M (2018). Unsavoury truth: How food companies skew the science of what we eat.

[R29] Oreskes N, Conway EM (2010). Merchants of doubt: How a handful of scientists obscured the truth on issues from tobacco smoke to global warming.

[R30] Pickering A, Pickering A (1992). Science as practice and culture.

[R31] Rossow I, McCambridge J (2019). The handling of evidence in national and local policy making: A case study of alcohol industry actor strategies regarding data on on-premise trading hours and violence in norway. BMC Public Health.

[R32] Savell E, Fooks G, Gilmore AB (2016). How does the alcohol industry attempt to influence marketing regulations? A systematic review. Addiction.

[R33] Stafford J, Kypri K, Pettigrew S (2020). Industry actor use of research evidence: Critical analysis of australian alcohol policy submissions. Journal of Studies on Alcohol and Drugs.

[R34] Stenius K, Babor TF (2010). The alcohol industry and public interest science. Addiction.

[R35] Stockwell T, Robert Solomon LM, O’Brien P, Vallance K, Hobin E (2020). Cancer warning labels on alcohol containers: A consumer’s right to know, a government’s responsibility to inform, and an industry’s power to thwart. Journal of Studies on Alcohol and Drugs.

[R36] Timmermans S, Tavory I (2012). Theory construction in qualitative research: From grounded theory to abductive analysis. Sociological Theory.

[R37] White J, Bero L (2010). Corporate manipulation of research: Strategies are similar across five industries. Stanford Law & Policy Review.

[R38] World Health Organization (2018). Global status report on alcohol and health 2018.

